# Epidemiology and management of wildlife contacts in an emergency department of French Guiana

**DOI:** 10.1371/journal.pntd.0013771

**Published:** 2025-12-03

**Authors:** Jules Maurer, Rémi Mutricy, Florian Negrello, Camille Deschamps, Mathieu Nacher, Hatem Kallel, Alexis Fremery

**Affiliations:** 1 Emergency Department, Cayenne General Hospital, Cayenne, French Guiana, France; 2 Emergency Department, Martinique University Hospital, Fort-de-France, Martinique, France; 3 French Guiana University, Cayenne, French Guiana, France; 4 CIC INSERM 1424, Cayenne General Hospital, Cayenne, French Guiana, France; 5 Intensive Care Unit, Cayenne General Hospital, Cayenne, French Guiana, France; Makerere University, UGANDA

## Abstract

**Context:**

The rapid population growth and urban expansion into the forests of French Guiana have increased human-wildlife encounters. In the Amazon, wild species are often perceived as hostile, and while serious injuries are rare, these encounters frequently lead to emergency department (ED) visits. This study aims to describe the epidemiology and management of human-wildlife contacts at the Cayenne ED.

**Materials and methods:**

A retrospective observational study was conducted from January 1 to December 31, 2019, including all wildlife contact cases treated at Cayenne Hospital ED. Data on patient context, clinical status, and treatment were collected.

**Results:**

In 2019, 402 patients presented to the ED for wildlife-related injuries. The median age was 32 years (16–49), and the M/F ratio was 1.7. Hymenoptera (26%) were the most common cause, followed by unidentified invertebrates (20%), snakes (16%), and scorpions (12%). Most injuries involved the lower limbs (43%). Anaphylaxis occurred in 4% of cases, neurological symptoms in 8%, and digestive issues in another 8%. Biological abnormalities were noted in 15% of patients. Ninety-two cases were classified as severe or at risk of worsening, predominantly involving snakes (49%), insects (42%), and scorpions (9%). Analgesics were given to 33%, antibiotics to 27%, and 11% were hospitalized. No deaths were recorded.

**Conclusion:**

In addition to snake and scorpion incidents, this study underscores the high frequency of Hymenoptera encounters. Despite an increase in wildlife contacts, morbidity and mortality remain low.

## Author summary

In French Guiana, rapid population growth and urban expansion are bringing people into closer contact with wildlife. Although serious injuries from these encounters are rare, they often lead people to seek emergency medical care. Our study looked at all wildlife-related injuries treated at the Cayenne Hospital Emergency Department in 2019. We found that insects like bees and wasps were the most common cause of injuries, followed by unidentified bugs, snakes, and scorpions. Most injuries involved the legs, and while some patients experienced severe reactions like anaphylaxis or neurological problems, most cases were not life-threatening. Snake bites, insect stings, and scorpion stings were the most serious types of encounters. About one in ten patients needed to be hospitalized, but there were no deaths. Our findings highlight the need for public awareness about the risks of wildlife encounters and the generally favorable outcomes with proper medical care. This information is important not just for healthcare providers but also for the general public living or traveling in areas where human-wildlife encounters are common.

## Introduction

French Guiana is a French overseas region located on the northeast coast of South America, bordered by the Atlantic Ocean, Brazil, and Suriname. Its 85,000 km^2^ territory is 96% covered by Amazonian forests and mangroves, which contain exceptional biodiversity [1]. French Guiana is experiencing significant demographic pressure, with its population having increased by 30% since 2007 to reach 300,000 in 2021 [2]. Leisure and professional activities but especially the growing need for urbanization has resulted in the intermingling of the Amazon jungle and inhabited areas, thereby increasing the number of human-wildlife encounters. Although these interactions rarely result in severe consequences [3], they are a frequent cause of emergency department visits and, in some cases, can lead to fatalities [4].

Nogalski et al. conclude that most hospitalisations or serious injuries caused by wildlife encounters are predominantly linked to contact with domestic animals [5]. In the study *Animal-Related Fatalities in the United States: An Update* [6], Langley RL reports that the number of deaths related to these encounters has risen since the 1990s, although the overall mortality rate has not changed. Currently, there are no precise data on wildlife encounters specific to the Amazon region. Certain species, however, are of particular interest to scientific and medical communities, with substantial data available on their epidemiology and management, especially concerning snakes and scorpions [7–9]. There are also broader studies, but these often include attacks by domestic animals, which are predominant and less specific to French Guiana’s unique context [10].

The primary objective of this retrospective observational study is to describe the epidemiological characteristics of wildlife-related encounters that led to emergency department visits in French Guiana. A secondary objective is to assess the initial medical management of these cases, with a focus on severity, treatment, and outcomes.

## Methods

### Ethics statement

This study qualifies as Research Not Involving Human Subjects (RnIPH) under both French and international regulations. It involved a retrospective review of pseudonymized data routinely collected during standard emergency care. In accordance with French law (Loi Jardé, decree no. 2016-1537), no ethics committee approval was required, as no interventional procedures were performed and no identifiable data were collected for research purposes. All data were extracted from medical records by authorized emergency department staff, under the supervision of the principal investigator. The study was declared to the hospital’s Data Protection Officer (DPO) and registered in the institution’s data processing register, in compliance with CNIL regulations. As per institutional policy, patients were informed collectively (via posters in the emergency department, the welcome booklet, and the hospital website) about ongoing research and their right to opt out. Any objections were fully respected.

### Study design

Given the lack of specific regional data, a retrospective review of emergency department records from the Cayenne Hospital in French Guiana was conducted from January 1, 2019, to December 31, 2019. All patients presenting to the Cayenne emergency department with a consultation reason or a diagnosis involving contact with a wild animal were included. Attacks by humans or contact with domestic animals were excluded from the study.

Initially, using the “keyword search” function of the emergency department’s computer software, we extracted from all the “diagnoses” and “consultation reasons” the data that met the criteria for contact with wildlife. We used the following keywords: “bite”, “sting”, “scratch”, “animal”, “insect”, “scorpion”, “snake”, “reptile”, “fish”, “ray”, “butterfly”, “wasp”, “bee”, “monkey”, “mammal”, “fauna”, etc. To ensure thoroughness, we also included word families (e.g., *bit, bites, bitten*, etc.).

### Data collection and abstraction

All relevant data were extracted from the medical records using a standardized data abstraction form developed prior to the review. The variables of interest were defined a priori. Data abstraction was performed by trained emergency department staff under the supervision of the principal investigator. In cases of incomplete or ambiguous information, consensus was reached through discussion within the study team. To ensure consistency, standardized procedures were applied throughout the data collection process. No formal interrater reliability testing was performed, but data consistency was verified during regular review sessions.

Data were all collected from medical records to address six main areas: population description, spatio-temporal circumstances of the contact, identification of the animal involved, description of the lesions and their potential biological consequences, pre-hospital and emergency department management, and patient outcomes.

### Definition of key variables

- Geographical location: The place of contact (urban vs. rural vs. forested area) was determined based on the patient’s description and the address in the medical record, when available. Areas were classified according to administrative zoning by French Guiana regional plans.
- Biological signs: These included any laboratory abnormalities indicating systemic involvement (e.g., thrombocytopenia, coagulopathy, renal or hepatic failure, rhabdomyolysis), based on local laboratory reference values. Specific thresholds for severity are detailed in the clinical criteria section below.
- School holidays: The calendar of French Guiana’s academic year 2019, as published by the Rectorat of Guyane, was used to identify school holiday periods. This allowed analysis of seasonal or activity-linked exposure.
- Weather conditions: Weather at the time of contact was approximated using the Meteo-France historical data for 2019, based on the date and location of the incident.
- Topography of lesions: Lesions were categorized by anatomical region (head/neck, trunk, upper limb, lower limb, multiple sites), based on the emergency physician’s physical examination notes and clinical documentation.

Based on our data, we identified a subgroup of patients considered at severe risk or at risk of deterioration, according to established criteria for hemorrhagic or septic shock, or anaphylaxis [11,12]. Clinical and biological signs were selected based on literature describing common complications from animal-related injuries including local and systemic inflammatory responses, allergic reactions, and organ dysfunction. These variables allowed us to stratify patient severity and assess clinical risk. A patient was classified as severe if they met at least one of the following criteria:

- Clinical data on admission: heart rate (HR) > 140/min, mean arterial pressure (MAP) < 65 mmHg, dyspnea with desaturation, or other signs of anaphylaxis (e.g., digestive symptoms).
- Management data: need for resuscitation maneuvers (e.g., fluid resuscitation, defined as administration of at least 500 mL of rapid intravenous crystalloids in adults or 10 mL/kg in children; use of catecholamines, sedation, intubation, or cardiopulmonary resuscitation).
- Laboratory data: fibrinolysis, defined by fibrinogenemia values below the laboratory standard and/or D-dimer values greater than 10 times the patient’s age, and/or prothrombin time (PT) reduced to less than 70%, not explained by another cause; acute renal failure, defined by increased creatinine values (above 100 µmol/L in women, 120 µmol/L in men, or based on pediatric estimates using the Schwartz formula); hepatocellular failure, defined as PT < 60%, not explained by another cause; rhabdomyolysis, defined by a significant increase in CPK (4 times above the laboratory standard).
- Monitoring data: need for continuous monitoring or admission to the intensive care unit.

### Statistical analysis

A descriptive analysis of the data was performed using Excel software (Microsoft, Redmond, WA, USA). Data are presented as numbers and percentages for qualitative variables, and as medians with interquartile ranges for quantitative variables.

## Results

### Characteristics of the population

In 2019, 696 cases related to contact with animals were recorded at the Cayenne Emergency Department, representing 1.4% of all admissions. Due to exclusion criteria, analyses were conducted on 402 cases involving contact with wildlife. All age groups were represented, with a median age of 32 years (16–49), and men were more frequently involved (n = 257, 64%). Among the patients included, 280 (88%) had no history of atopic or allergic conditions (excluding missing data).

### Characteristics of contacts

**Fig 1:**
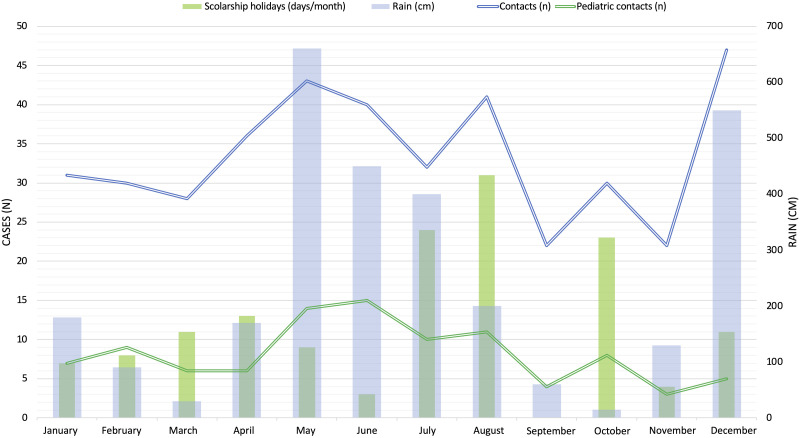
Emergency department consultations following contacts with wild animals during the year.

**Fig 2:**
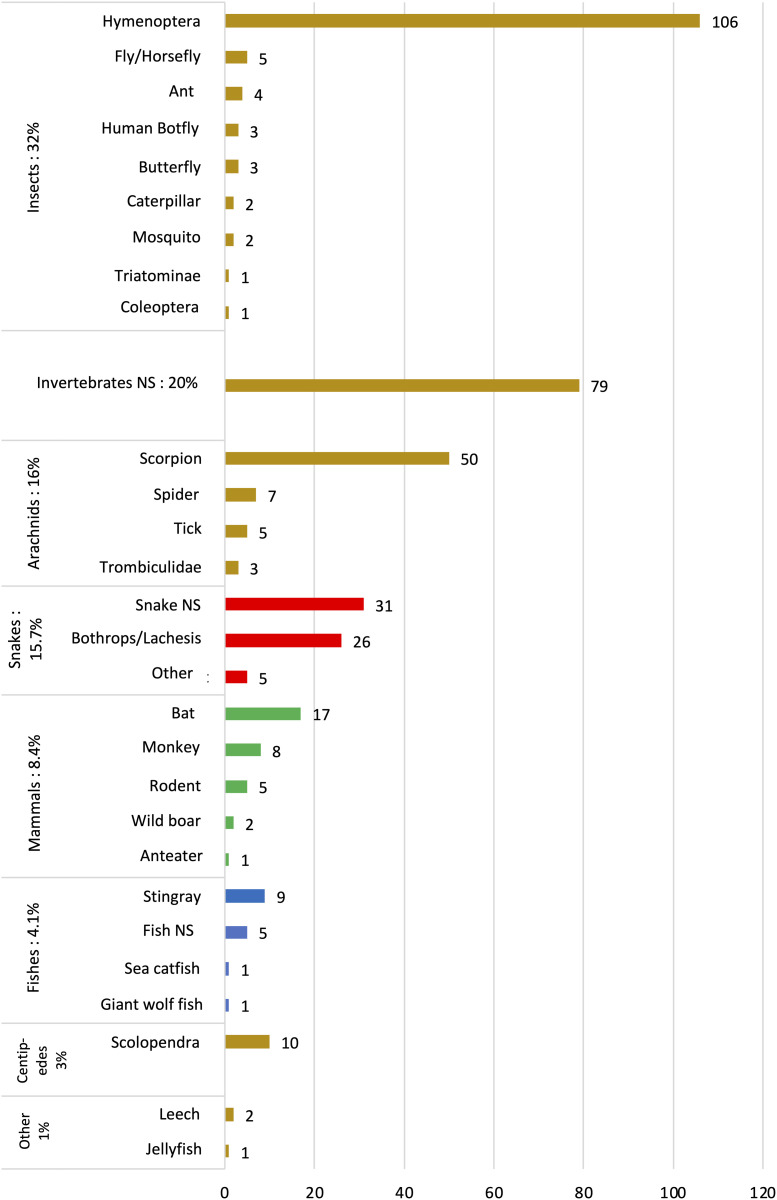
Human-wildlife contacts by class (n). NS, not specified.

The circumstances of the contacts are summarized in Table 1. Most of the encounters occurred in forest areas or near a river (n = 81, 57%). The distribution of contacts throughout the year, along with their correlation to rainfall periods, is shown in Fig 1. The animals most frequently involved in consultations were insects (n = 127, 32%). For 79 patients (20%), the aggressor was a presumed invertebrate that could not be identified by either the patient or the healthcare provider. The various classes of animals involved are depicted in Fig 2. The most frequently identified species were Hymenoptera (106 cases, or 27%).

**Table 1:** Circumstances of human-wildlife contacts.

	Total	Mammals n = 33	Snakes n = 62	Fishes n = 16	Centipedes n = 10	Scorpions n = 50	Hymenoptera n = 106	Other invertebrates n = 79
Geographic locations (n = 143)								
Forest/River	81 (57%)	12 (71%)	29 (73%)	9 (75%)	–	6 (35%)	15 (65%)	10 (33%)
Beach/Sea	6 (4%)	1 (6%)	–	3 (25%)	–	–	–	2 (7%)
Home/Garden	49 (34%)	3 (18%)	10 (25%)	–	4 (100%)	11 (65%)	6 (26%)	15 (50%)
Public space	7 (5%)	1 (6%)	1 (3%)	–	–	–	2 (9%)	3 (10%)
Day hours (n = 256)	200 (78%)	2 (11%)	3 (6%)	–	3 (43%)	30 (71%)	78 (94%)	32 (80%)

### Lesions topography

**Fig 3:**
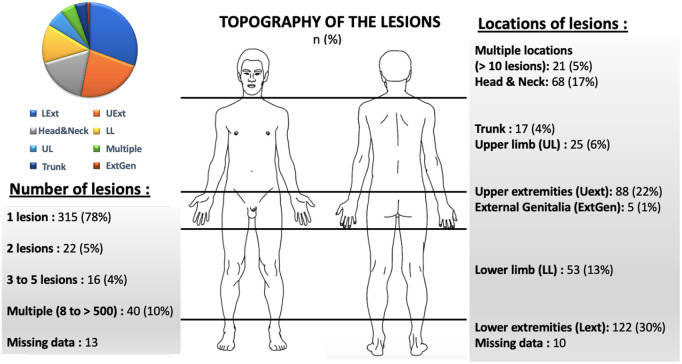
Topography of lesions n (%). Modified from a free image obtained on openclipart (https://openclipart.org/detail/314881/human-male-and-female-body-line-art).

The topography of the lesions is shown in Fig 3. Most patients had a single skin lesion (n = 315, 78%). Lesions were predominantly localized on the upper and lower limbs, consistent with typical exposure sites in environmental settings. However, 21 patients (5%) presented with multiple lesions (>10). Lesions were most commonly found on the extremities of the limbs.

### Clinical and biological signs

Biological tests were performed in 126 patients (31%), of whom 62 (49%) presented with at least one of the following abnormalities: fibrinolysis, hepatocellular failure (HCI), biological inflammatory syndrome (BIS), acute renal failure (ARF), and/or rhabdomyolysis. The clinical and biological signs observed are reported in Table 2.

**Table 2:** Clinical and biological signs according to the animal class involved.

Variable	Total	Fishes n = 16	Snakes n = 62	Mammals n = 33	Hymenoptera n = 106	Scorpions n = 50	Centipedes n = 10	Others n = 124
Facial oedema (n = 385)	53 (14%)	–	–	1 (3%)	41 (43%)	–	1 (10%)	10 (8%)
Urticaria (n = 365)	45 (12%)	–	–	1 (3%)	31 (35%)	–	1 (11%)	12 (10%)
Digestive symptoms (n = 327)	26 (8%)	1 (6%)	1 (2%)	–	20 (22%)	2 (9%)	–	2 (2%)
Pus/abscess (n = 395)	29 (7%)	2 (13%)	1 (2%)	1 (3%)	2 (2%)	–	–	23 (19%)
Dyspnea (n = 398)	15 (4%)	–	–	–	10 (10%)	–	–	5 (4%)
**Anaphylaxis (n = 392)**	17 (4%)	–	–	–	12 (11%)	–	–	5 (4%)
Necrosis (n = 399)	11 (3%)	–	8 (13%)	–	1 (1%)	–	–	2 (2%)
Neurologic symptoms (n = 399)	8 (2%)	–	–	–	–	8 (18%)	–	–
Decaying wound (n = 399)	4 (1%)	1 (6%)	–	2 (6%)	–	–	–	1 (1%)
Dysphonia (n = 393)	1 (0%)	–	–	–	1 (1%)	–	–	–
Shock (n = 399)	–	–	–	–	–	–	–	–
***Laboratory abnormalities***	**n = 126**	**n = 3**	**n = 57**	**n = 4**	**n = 20**	**n = 28**	**n = 0**	**n = 14**
Fibrinolysis	29 (28%)	–	29 (73%)	–	–	–	–	–
Hepatic failure	29 (27%)	–	26 (63%)	–	–	1 (4%)	–	2 (3%)
Inflammatory syndrom	19 (16%)	–	8 (16%)	1 (25%)	4 (20%)	–	–	6 (43%)
Kidney failure	32 (29%)	–	14 (31%)	–	14 (73%)	3 (12%)	–	1 (3%)
Rhabdomyolisis	6 (8%)	–	4 (7%)	–	1 (5%)	1 (4%)	–	–

Percentages are calculated row-wise for each variable, based on the number of patients in whom the variable was documented. Missing data are excluded.

### Patients presenting severe criteria

A total of 92 patients (23%) met the criteria for being severe or at risk of worsening, as outlined earlier. The animals involved in these cases were exclusively snakes (n = 46, 50%), insects (n = 39, 42%), and scorpions (n = 7, 8%). Biological tests were performed on 73 (80%) of these patients, and 69 (76%) showed abnormalities. None of the patients met the criteria for shock. Viperidae were responsible for 26 (94%) of the serious snake bites where the species was identified. Of the 29 patients (63%) with signs of fibrinolysis, 28 (96%) received at least one dose of specific antivenom. The only case of a scorpion sting resulting in both rhabdomyolysis and hepatocellular failure involved a 5-year-old child. Among the serious cases of Hymenoptera stings (n = 29, 31%), 26 (90%) were stung on the head or neck, 16 (55%) received more than 20 stings, and 12 (41%) showed signs of anaphylaxis. Although “shock” was one of the predefined severity criteria, no patient fulfilled the necessary clinical or biological thresholds.

### Patient management

The therapeutic management of patients is detailed in Table 3. The animal class most frequently requiring tier 3 analgesics was fish and rays (n = 4, 25%). A total of 107 patients (27%) received antibiotic treatment, with 92 (86%) being treated with amoxicillin-clavulanic acid. Nearly half of the patients whose attacker was an unknown invertebrate received antibiotics, although only 9 (23%) presented with local signs of infection. Among the snake-bitten patients treated with antibiotics, 22 (92%) received amoxicillin-clavulanic acid, while only 2 (8%) were treated with third-generation cephalosporins (C3G). Among patients presenting with signs of anaphylaxis, only 7 (6%) received injectable adrenaline, and 6 (5%) received crystalloid infusion. Only one patient required deep sedation and orotracheal intubation due to dysphonia following anaphylaxis from a wasp sting. There were no deaths.

**Table 3:** Treatments of injuries.

Treatments	Total	Fishes n = 16	Snakes n = 62	Mammals n = 33	Hymenoptera n = 106	Scorpions n = 50	Scolopendra n = 106	Others n = 120
IV infusion	107 (27%)	4 (25%)	46 (72%)	3 (9%)	29 (27%)	14 (28%)	1 (10%)	10 (8%)
IV ressuscitation	20 (5%)	–	4 (6%)	–	15 (14%)	1 (2%)	–	–
Antihistamine	147 (37%)	1 (6%)	3 (5%)	1 (3%)	91 (86%)	3 (6%)	6 (60%)	43 (35%)
Corticoids	79 (20%)	–	2 (3%)	–	53 (50%)	3 (6%)	3 (30%)	18 (15%)
Analgesic level 3	24 (6%)	4 (25%)	11 (17%)	–	5 (5%)	6 (12%)	–	–
Analgesic level 2	28 (7%)	2 (13%)	9 (14%)	1 (3%)	2 (2%)	6 (12%)	5 (50%)	2 (2%)
Analgesic level 1	80 (20%)	3 (19%)	11 (17%)	1 (3%)	18 (17%)	9 (18%)	2 (20%)	34 (28%)
Antibiotics	109 (27%)	9 (56%)	24 (38%)	21 (62%)	8 (8%)	1 (2%)	3 (30%)	41 (34%)

IV: intravenous.

## Discussion

This 2019 study analyzed contacts between humans and wildlife that required treatment at Cayenne’s emergency departments. More than 400 cases were treated, accounting for 0.8% of emergency consultations. No deaths were recorded. Most victims (n = 310, 77%) did not meet the severity criteria, and only 44 patients (11%) were admitted to hospital. However, the number of admissions to intensive care was relatively high, primarily due to the systematic hospitalisation of patients bitten by viperidae with envenomation, following the recommendations of Resiere et al. [13]. Despite the significant number of consultations related to wildlife contact in French Guiana, serious cases and fatalities remain rare, consistent with findings from other studies in the literature [14]. Unlike data from Western countries [15], contacts with wildlife in French Guiana predominantly occur in rural and forested areas, aligning with previous research conducted in French Guiana and Brazil [14,16].

Our study also highlighted that contact with Hymenoptera was the most frequent type of encounter (n = 106, 27%), similar to trends seen in temperate Western regions [5]. However, this contrasts with neighboring Brazil, where scorpion (30%) and snake (25%) encounters are more common [14].

The second key aspect of this study was the evaluation of patient management. Pain was a frequent reason for consultation, necessitating the use of analgesics in over a third of cases. Analysis of the infectious risks associated with wildlife encounters revealed that antibiotics were widely prescribed, even in cases where lesions were minor—100% of wound cases treated with antibiotics showed no local signs of infection (n = 85, 77%). Further studies are warranted to assess the appropriateness of antibiotic prophylaxis versus a more clinical observation-based approach, or to justify expanding the use of antibiotics in the humid tropical context [17–20].

Moreover, the management of Hymenoptera stings in French Guiana requires further examination [21]. Treatment approaches varied: only 7 out of 17 patients who experienced an allergic reaction received an early adrenaline injection, and 6 patients received vascular filling. Given the frequent occurrence of Hymenoptera stings in French Guiana, specific studies could raise clinician awareness and explore targeted treatments, such as anti-venoms [21]. Further research is particularly needed for cases involving multiple stings, which may lead to envenomations with clinical presentations ranging from anaphylactoid to non-anaphylactic reactions [22,23]. Additionally, geographical distance and delays in accessing care must be considered to ensure appropriate management of wildlife encounters in remote regions [24].

This study updates data on human-wildlife contact in French Guiana and identifies several areas for improvement in patient management. Expanding data collection to include all hospitals in French Guiana would provide more comprehensive and exhaustive data for the region. One crucial issue is the lack of specialists capable of identifying the species involved. The implementation of precise species coding and the creation of a registry for monitoring envenomations, alongside the establishment of a toxicovigilance unit, would enhance species identification and improve the management of wildlife-related injuries.

Lastly, this study chose not to address inoculation and vector-borne diseases or other zoonoses. However, French Guiana faces frequent tropical epidemics, which, unlike direct attacks by wildlife, present significant morbidity and mortality risks. Numerous studies in recent years have highlighted the efforts of public health authorities to combat these deadly diseases, which are indirectly linked to wildlife proximity [25,26].

This study is limited by its retrospective design and potential information bias related to incomplete or missing data. However, the large sample size, systematic data abstraction method, and inclusion of both clinical and biological severity criteria help provide a valuable overview of wildlife injuries in this region.

## Conclusion

This study provides a general overview of wildlife-related contacts treated in the Cayenne Hospital Emergency Department. In addition to the well-studied snake and scorpion attacks from recent years, this study highlights the prevalence of Hymenoptera stings, which require further in-depth research. However, it remains necessary to supplement this analysis with data from across the entire country.
